# The origins and spread of the opium poppy (*Papaver somniferum* L.) revealed by genomics and seed morphometrics

**DOI:** 10.1098/rstb.2024.0198

**Published:** 2025-05-15

**Authors:** Rui S. R. Machado, Vincent Bonhomme, Raül Soteras, Angele Jeanty, Laurent Bouby, Allowen Evin, M. Joao Fernandes Martins, Sandra Gonçalves, Ferran Antolín, Aurélie Salavert, Hugo Rafael Oliveira

**Affiliations:** ^1^ ICArEHB, Interdisciplinary Center for Archaeology and Evolution Human Behaviour, Universidade do Algarve, Campus de Gambelas, Faro, Portugal; ^2^ ISEM, Université Montpellier, CNRS, IRD, EPHE, Montpellier, France; ^3^ Division of Natural Sciences, German Archaeological Institute (DAI), Berlin, BE, Germany; ^4^ UMRAASPE/BIOARCH, Muséum National d'Histoire Naturelle (MNHN), Centre National de Recherche Scientifique (CNRS), Alliance Sorbonne Université, Paris, France; ^5^ MED – Mediterranean Institute for Agriculture, Environment and Development & CHANGE – Global Change and Sustainability Institute, Faculdade de Ciências e Tecnologia, Universidade do Algarve, Campus de Gambelas, Faro, Portugal; ^6^ Environmental Sciences, Integrative Prehistory and Archaeological Science (IPNA/IPAS), University of Basel, Basel, Switzerland; ^7^ Faculdade de Ciências Humanas e Sociais, Universidade do Algarve, Campus de Gambelas, Faro, Portugal

**Keywords:** domestication, opiates, agrobiodiversity, plant genome, seed morphology

## Abstract

The opium poppy (*Papaver somniferum* L.) is one of the most important plants in human history. It is the main source of opiates used as analgesic medicines or psychotropic drugs, the latter related to addiction problems, illegal trafficking and geopolitical issues. Poppyseed is also used in cooking. The prehistoric origins, domestication and cultivation spread of the opium poppy remain unresolved. Traditionally, *Papaver setigerum* has been considered the wild ancestor with early cultivation presumed to have occurred in the Western Mediterranean region, where *setigerum* is autochthonous. Other theories suggest that *somniferum* may have been introduced by Southwest Asian early farmers as a weed. To investigate these hypotheses, we analysed 190 accessions from 15 *Papaver* species using genotype-by-sequencing and geometric morphometric (GMM) techniques. Our analysis revealed that *setigerum* is the only taxa genetically close to *somniferum and can be better described as a subspecies*. The domesticated plants are, however, distinct from *setigerum*. Additionally, GMM analysis of seeds also revealed morphological differences between *setigerum* and *somniferum*. Some phenotypically wild *setigerum* accessions exhibited intermediate genetic features, suggesting introgression events. Two major populations were found in *somniferum* and, to some extent, these correspond to differences in seed form. These two populations may reflect recent attempts to breed varieties rich in opiates, as opposed to varieties used for poppyseed production. This study supports the idea that opium poppy cultivation began in the Western Mediterranean, with *setigerum* as the wild progenitor, although some wild varieties are likely to be feral forms, which can confound domestication studies.

This article is part of the theme issue ‘Unravelling domestication: multi-disciplinary perspectives on human and non-human relationships in the past, present and future’.

## 1. Introduction

The opium poppy (*Papaver somniferum* L.), henceforth referred to as *somniferum*, is one of the most famous and infamous plant species in history. Its latex has a high content in benzylisoquinoline alkaloids (BIAs), such as morphine, codeine and noscapine, which give this species its medicinal and narcotic properties. The opiates produced are some of the most powerful naturally occurring painkilling substances, although their side effects include tolerance and addiction. In the past 2500 years, it was presumably cultivated for the production of opium and its derivative opiates (i.e. heroin), used as anaesthetic and as a mind-altering substance, with profound implications for past and present societies [1]. *Somniferum* seeds were also used in some culinary cultures (e.g. poppy seed bread) and as source of oil [2,3]. Presently, besides being used as an ornamental flower, *somniferum* is grown by the pharmaceutical and food industries, with an estimated worldwide area of 27 773 ha for poppyseed, and an estimated 3 16 000 ha for illegal cultivation in 2022 [4,5].

For such an economically and culturally important species, little is known about its origins. Its taxonomical classification as species or subspecies is not consensual, and the World Flora Online reports the name is currently unchecked and awaiting taxonomic scrutiny (wfo-0001367210). Based on cytological analysis, *Papaver setigerum* DC. (henceforth referred as *setigerum*) is considered the wild progenitor of the cultivated *Papaver somniferum* [6,7]. *Setigerum* is sometimes classified as subspecies *P. somniferum* subsp. *setigerum* (DC.) Arcang. The main differences between the wild and domesticated opium poppy are the larger capsule and seed size, retention of seeds and higher levels of morphine in the latter [7,8]. Its domestication is thought to have occurred in the Western Mediterranean area, where *setigerum* is naturally distributed today [6,9]. This hypothesis is corroborated by archaeobotany. Although undated remains from the Balkans and Israel were attributed to more ancient periods, the earliest securely identified and radiocarbon dated *setigerum*/*somniferum* seeds were found in an archaeological context dated to *ca* 5500 BCE in Central Italy, associated with the first farming communities there [10]. If so, poppy was the only plant species domesticated in Europe during the Neolithic [11], as the cereals and legumes cultivated by the first farmers originated in Southwest Asia and were domesticated between 10 and 7000 BCE [12].

Alternatively, *somniferum* could have been introduced as a weed of cereal crops and exploited exclusively in the Western Mediterranean where Neolithic farmers recognized its uses. Segetal weeds that would become crops include rye and oat [13]. In this scenario, the wild progenitor could be one of the many wild *Papaver* species that occur in Southwest Asia, with the Western Mediterranean *setigerum* being a feral form. Feral forms resembling wild forms can mislead domestication studies, as recently recognized [14,15]. Feral forms mimicking wild progenitors have been reported for barley, rice, wheat, eggplant, radish and brassicas [16–22]. The feral nature of at least some *setigerum* populations is supported by the occurrence of both diploid (*2n* = 2X = 22 chromosomes) and tetraploid (*2n* = 4X = 44) types [7,23,24]. The mechanisms of formation of polyploid hybrids in plants are well known and occur frequently [23–26]

After domestication, the spread of *somniferum* cultivation in Europe was very fast (<200 years, based on radiocarbon dating of poppy seeds). The introduction of agriculture in Europe from its Fertile Crescent origins followed two routes after its introduction in the Balkans, each characterized by a distinctive material culture, especially in pottery types: the Cardial/Impressa Culture along the Mediterranean shores, and the *Linearbandkeramik* (LBK) Culture, following the main rivers into Central and Northern Europe [27]. The fast spread of *somniferum* is intriguing as these two cultures are usually thought of as separated, although this is being reconsidered by some scholars [28]. Poppy is found on Neolithic archaeological sites located outside the *setigerum* distribution area, as early as the arrival of the first agro-pastoralists of the LBK culture, i.e. around 5200 cal BC, and even in the western Alps, as early as 5000 cal BC [10]. One explanation for this is that *somniferum* was domesticated in the Western Mediterranean by people of the Impressa/Cardial culture and then exchanged with people from the Central European LBK culture. Or, if it were a weed brought with cereals from Southwest Asia or the Balkans, both early farming cultures could have started cultivating it independently. The routes by which *somniferum* cultivation spread in the Old World are unknown, although some first steps have been taken using geometric morphometric analysis of archaeological seeds [9].

When combined with archaeology, the genomic analysis of extant modern wild populations and landrace varieties can elucidate a crop domestication process [29,30]. Questions such as ‘*which wild progenitor’, ‘where’, ‘how many times’* or ‘*how fast*’ can be addressed by looking at whole-genome differences between accessions, determining which wild populations are genetically more similar to modern domesticated plants and looking at the geographical distribution of genetic diversity. It also allows the identification of genes underlying the domestication syndrome traits [31]. These have not been identified in *somniferum* yet. Likewise, mapping the geographical distribution of genetic diversity in this crop can reveal the routes by which its cultivation was introduced. Genotype-by-sequencing (GBS) is a genome complexity reduction method that allows single-nucleotide polymorphisms (SNPs) to be found in a panel of geographical and taxonomical diverse accessions. GBS has been used to investigate the domestication process of major crops such as lentil [32], potato [33], rice [34] and maize [35]; as well as lesser ones, for example pepper [36], cabbages [37], eggplant [38] and crotalaria [39].

The genome of *somniferum* has been sequenced, and the species’ population structure in a global panel of accessions has been recently investigated [40–42]. Most ‘-omics’ technologies work on this species has focused on the morphine production pathways and its evolution [43–47]. To our knowledge, using genomics to investigate the history of *somniferum* domestication has not been attempted. Phylogenetic studies of the Papaveraceae family based on both chloroplast and nuclear markers consistently place *setigerum* in the same clade as *somniferum* [6,48–52]. These are, however, based on a handful of accessions and have not investigated the possibility of pollen and genetic exchange (introgression) between species.

Geometric morphometrics (or ‘modern’ morphometrics, further abbreviated GMM) provide a mathematical approach and the statistical framework to measure, describe and study the variability of form, which can be decomposed into shape and size [53,54]. In archaeobotany, the use of GMM has significantly advanced the ability to document, understand the emergence of cultivated and/or domesticated plants across both time and space, despite the fact that this material is often altered by post-depositional processes [53–57]. A recent seminal study by Jesus *et al*. [9] showed that *Papaver* species could be reliably identified, a method that has been further applied to archaeological contexts. We expand that work by comparing several *setigerum* and *somniferum* modern accessions, to investigate if genetic clusters match seed shape variation, revealing a link between genotype and seed phenotype.

Here, we investigate the origins and spread of opium poppy by complementing the archaeobotanical framework with a GBS and GMM study of 190 present-day *Papaver* accessions, belonging to 15 taxa and 38 present-day countries. This is a novel methodological approach that brings a multidisciplinary perspective to the evolution of this species. Moreover, the data generated can be used for plant breeding of new poppy varieties. We address the following questions: (i) *which species was somniferum’s* (opium poppy) *wild progenitor*; (ii) *where was the poppy domesticated;* (iii) *and how many times;* (iv) *how its cultivation spread;* (v) *which traits underlie the domestication syndrome in this species*. The data are interpreted in the context of archaeological, historical and botanical proxies. To our knowledge, it is the first attempt to use genomics (on its own and combined with seed GMM) to elucidate the origins of the opium poppy.

## 2. Methods

### (a) Plant materials

A set of 190 wild and cultivated accessions within the *Papaver* genus (Papaveraceae) were selected (electronic supplementary material, S1). We included as many wild species and accessions as available in seed banks to detect ancestry relationships or gene flow with cultivated *somniferum* (number of accessions of each species under brackets): *Papaver armeniacum* (1), *Papaver dubium* (3), *Papaver fugax* (1), *Papaver glaucum* (3), *Papaver hybridum* (1), *Papaver macrostomum* (1), *Papaver nudicale* (1), *Papaver occidentale* (2), *Papaver orientale* (8), *Papaver pseudo-orientale* (2), *Papaver rhoeas* (4), *Papaver rupifragum* (1), *Papaver umbonatum* (4) and *Papaver setigerum* (19). *Papaver occidentale* has recently been re-classified as *Papaver alpinum* or *Oreomecon alpina* Banfi [58]. For *setigerum,* we sampled all countries from where seeds were collected and stored in germplasm banks (France, Italy, Portugal and Spain). To these, we added 139 *P. somniferum* accessions from Eurasia and North Africa, including landraces/heirloom varieties (116) and cultivars/commercial breeds (23). Seeds were ordered from USDA-GRIN (United States), IPK (Germany), Nordgen (Sweden), CRI-Prague (Czechia) and Centro de Recursos Fitogenéticos (Spain). *Papaver occidentale* DNA was kindly provided by Christian Parisod (University of Fribourg). Seeds were vernalized for four weeks at 4°C and sown in pots filled with a mixture of vermiculite and perlite. Plants were grown in a room under constant temperature (25 ± 2°C) and a photoperiod of 16 h/8 h (light/dark). DNA was extracted from fresh leaves pulverized with liquid nitrogen using the DNeasy Plant Mini Kit (Qiagen). For some accessions, a bulk of 10−15 seeds was pulverized inside a tin foil wrap, to compare with individual leaves from the same accession. DNA was quantified with a Qubit dsDNA HS assay on a Qubit 2.0 Fluorometer. DNA extractions were run on a 1% agarose gel to test for integrity. Two 96-well plates were prepared, with each well containing between 100 and 300 ng of DNA. Seeds of 153 of these accessions of *setigerum* and *somniferum* were used for the GMM analyses.

### (b) Genotype-by-sequencing

#### (i) Library preparation and sequencing

Genotyping-by-sequencing was performed by LGC Genomics GmbH (Berlin, Germany). Libraries were prepared using a combination of PstI-MseI restriction enzymes with an insert size mean range of 210 bp. After indexing, libraries were pooled and sequenced in an Illumina NovaSeq 6000 platform (150 bp paired-end reads). The raw sequencing reads were deposited in the BioProject database of NCBI (https://www.ncbi.nlm.nih.gov/bioproject/PRJNA1160476).

#### (ii) Read pre-processing

Demultiplexing of barcoded samples for each sequencing lane and library groups into samples according to their inline barcodes, with subsequent verification of restriction site, were done using Illumina bcl2fastq software (one or two mismatches or Ns were allowed in the barcode read when the barcode distances between all libraries on the lane allowed for it; no mismatches or Ns were allowed in the inline barcodes, but Ns were allowed in the restriction site). Clipping of sequencing adapter remnants from all reads and further trimming was done with CUTADAPT v. 3.2 [59]. Reads with a final length <20 bases were discarded, as well as reads with 5′ ends not matching the restriction enzyme site. Reads were trimmed at the 3′-end to get a minimum average Phred quality score of 20 (over a window of 10 bases), and those containing Ns were removed.

#### (iii) Genotype-by-sequencing alignment and single-nucleotide polymorphism discovery

Quality-trimmed reads were aligned against the *somniferum* reference genome (genome assembly ASM1011999v1, cultivar Roxanne, GCA_010119995.1) using BWA-MEM v. 0.7.12 producing one combined alignment for all samples in a coordinate-sorted Binary Alignment Map (BAM) format [60,61]. Variant discovery and genotyping of samples were carried out with Freebayes v. 1.2.0 [62] with the following specific parameters being used (--min-base-quality 10 --min-supportingalleleqsum 10 --read-mismatch-limit 3 --min-coverage 5 --no-indels --min-alternatecount 4 --exclude-unobserved-genotypes - -genotype-qualities --ploidy 2 --nomnps --nocomplex --mismatch-base-quality-threshold 10). Filtering of variants using a GBS-specific rule set to produce Variant Call Format (VCF) files was carried out using proprietary pipelines (LGC Genomics Ltd) using the following parameters: the read count for a locus must exceed 8 reads, genotypes must have been observed in at least 66% of samples and minimum allele frequency across all samples must exceed 5%. On a separate analysis that included only *P. somniferum* and *setigerum*, the ploidy parameter was set to --ploidy 4 to account for the possibility of tetraploid *setigerum* in the set [23]. Downstream analysis of VCF files was done with TASSEL 5 v. 5.2.74 [63] and VCFtools v0.1.16 [64].

### (c) Population structure and phylogenetic inference

To find genomic relationships between the 190 accessions and describe the population structure in the whole panel (267 903 SNPs), we undertook a principal component analysis (PCA). The PCA function in TASSEL 5 was used. We also used the R packages vcfR v. 1.14.0 [65], adegenet v. 2.1.10 [66] and ggplot2 v. 3.4.3 [67], within the R programming language integrated development environment RStudio v. 2023.06.2+561 [68,69]. Four accessions (PI304971, C42, D52 and PI688271) were excluded from further analysis for having more than 95% missing data. In an exploratory analysis, different filters for missing data and minor allele frequency (MAF) were tested. PCA was repeated for the set of 186 accessions including the different wild *Papaver* species (accept SNPs with no data in 10% of the samples, MAF = 0.05 and 4102 SNPs), henceforth referred to as Dataset1*.* Dataset1 was not thoroughly filtered to exclude SNPs with any missing data as the presence of different species significantly reduced the number of SNPs detected in all individuals (only 162 SNPs would be retained if the set had no missing data).

Another set containing only *somniferum* and *setigerum* (158 accessions, no missing data, MAF = 0.05 and 7048 SNPs) was produced, henceforth referred as Dataset2. This was done considering the original VCF file and retaining only the accessions belonging to these two taxa (assuming diploidy). Additionally, a VCF file containing only *somniferum* and *setigerum* accessions but assuming tetraploidy was produced and filtered (no missing data, MAF = 0.05 and 5771 SNPs). This was Dataset3.

Population structure was also examined with parametric methods. The Bayesian model-based clustering algorithm STRUCTURE 2.3.4 [70] was run with *K* values between 1 and 20, with a 50 000 burnin, 100 000 Markov chain Monte Carlo (MCMC) iterations and 10 independent runs for each value of *K*. The most likely *K* values were chosen based on the Δ*K* method [71], using the online version of STRUCTURE HARVESTER [72]. STRUCTURE was run for the two abovementioned datasets: with 186 and 158 accessions, respectively. The former aimed to investigate relationships and possible gene flow across genus *Papaver*, and the latter to look at *somniferum* domestication process. Q matrixes were plotted in MS Excel, and the geographical distribution of the different STRUCTURE-defined clusters mapped using the open source QGIS (QGIS Association) and the R package rgdal [73], with gstat for spatial interpolation [74].

In TASSEL 5, nucleotide diversity per base pair (π) [75] and Tajima’s *D* [76] were calculated along each chromosome of *P. somniferum* and *P. setigerum* samples based on Dataset2, using a window size of 30 SNPs and a gap of 10. Pairwise Nei genetic distance and Pairwise *F*_ST_ between taxa and between individual accessions were calculated with packages adegenet and vcfR [65,77]. Genetic diversity measures (*H*_O_ and *H*_E_) for species and populations defined by STRUCTURE (to describe the distribution of diversity between taxa, populations and individuals), were calculated in adegenet.

For phylogenetic analysis, a maximum-likelihood (ML) method was used in RAxML v. 8.0.0 [78]. The python script vcf2phylip.py v. 2.0 [79] was used to convert .vcf files to phylip format. We determined the best-fit evolutionary model using jModelTest v. 2.1.4 [80]. For the 186 accessions of Dataset1, the general time-reversible model of nucleotide substitution including gamma-distributed rates across sites (GTR + Γ) was chosen. The best ML tree was obtained by coupling 100 rapid bootstrap iterations and searching for the best-scoring ML tree in a single randomized axelerated maximum likelihood (RAxML) run. For *somniferum* accessions MrBayes v. 3.2.7 a [81] was also used. Two independent runs, with four chains and 1 million generations each, were computed using MrBayes. Markov chains were sampled every 500 generations with swaps of states between chains being tried on each generation of the run. The burnin was set to 25%. MrBayes was conducted at the CIPRES Science Gateway v. 3.34.

### (d) Detecting introgression and selection

The ABBA–BABA test was used to investigate introgression between *setigerum* and *somniferum* accessions [32,82]. In this case, individuals suggested by STRUCTURE and PCA to be putative hybrids or misclassified accessions were excluded. Dsuite v. 0.1 was employed on Dataset2 to calculate the *D* statistic [83]. *Papaver orientalis* was used as an outgroup.

Genome-wide association study (GWAS) was performed on Dataset2 to detect SNPs associated with (i) seed colour and (ii) domesticated status (in this case, ‘domestication’ is considered a single trait). In the former, the predominant colour of seeds was recorded. Four colours were considered: blue, brown, dark brown and white. In the latter, two traits were considered: ‘domesticated’ or ‘wild’. In TASSEL 5, the first five components of a PCA (population structure) and a kinship matrix (Q + K model) were used to calculate a mixed linear model (MLM). A general linear model (GLM) was also computed. A Bonferroni correction with *α* = 0.001 was used as threshold for marker–trait associations. The flanking sequences of SNPs detected were submitted to a BLAST search using the standard nucleotide BLAST tool at the NCBI platform (https://blast.ncbi.nlm.nih.gov/Blast.cgi).

To find genetic signals of environmental adaptation in *somniferum*, associations between allele frequencies in Dataset2 and 19 environmental proxies were tested (https://www.worldclim.org/data/bioclim.html), using the R package gradientForest v. 0. 1−37 [84]. Only accessions with available latitude and longitude information provided by the germplasm banks were considered. Environmental variables were extracted for the provenance location of each accession using bioclimatic layers available in the WorldClim 2.1 database [85]. We extracted the 19 BIO variables at 30-arcsecond resolution (approx. 1 km^2^). Environmental data were extracted using the raster v. 3.6−26 package in R [86], following the script in https://www.benjaminbell.co.uk/2018/01/extracting-data-and-making-climate-maps.html. To validate our analyses, we compared the results with those run from randomized matches between BIO and Elev data (geographical coordinates were maintained) and allele frequency data. Relationships between pairwise distances (geographical, environmental and genetic) were tested with a series of Mantel tests [87]. Pairwise genetic distances were calculated in TASSEL 5.0, whereas pairwise geographical distances were calculated using the package geosphere v. 1.5−18 [88] and the haversine distance option. The pairwise environmental distances were calculated as Euclidean distances using the vegan package 2.6−4 [89].

### (e) Geometric morphometrics of seeds

Seeds from 153 accessions in Dataset2 (*somniferum* and *setigerum*) were photographed using a Hirox RH-2000, following the procedures reported in Jesus *et al*. [9]. For each accession, 18−35 seeds were photographed, resulting in a total of 4857 images. Seed outlines were normalized by recording the coordinates of five landmarks using software ImageJ [90]. Measurement error minimization and improvement of reproducibility were carried out as shown elsewhere [9]. Seed shape was analysed using outline analysis based on elliptic Fourier transforms (EFTs) using Momocs 1.4.1 [91] in the R 4.1.3 environment [69]. Outlines were normalized for position, size and orientation using full generalized Procrustes alignment, with landmark two set as the starting point for each outline. EFTs were calculated from 720 evenly spaced points along the curvilinear abscissa. Five harmonics, previously shown to be a good trade-off between accuracy of shape capture and measurement error [9], were used, yielding 20 EFT coefficients per view (four per harmonic), along with length, width and cell count as morphometric variables.

To explore overall shape variability, PCA was calculated on the full matrix of Fourier coefficients, with the first two principal components used as synthetic shape variables. Linear discriminant analyses (LDA) with leave-one-out cross-validation were employed to train and evaluate classification models. To account for unequal sample sizes among groups, the ‘permutation balanced LDA’ approach was employed [92]. A multivariate linear model was used to test for associations between shape and colour of seeds. Additionally, associations between morphometrical data and the population genetic assignations for each accession in the STRUCTURE analysis (see above) were investigated using 100 LDA iterations (100 permutations of balanced datasets) and multivariate analysis of variance.

## 3. Results

### (a) Genotype-by-sequencing

The combination of PstI-MseI restriction enzymes with DNA from 190 wild and cultivated *Papaver* accessions resulted in a total of 288 million reads (71.8 Gb of data). Mapping of reads against the *somniferum* reference genome was met with a mapping rate of 99.5% (the total number of SNPs across all samples with a minimum read count of 8 was 267 903). Using a pipeline containing only *P. somniferum* and *P. setigerum* accessions and assuming tetraploidy, less SNPs were detected (185 566).The average number of quality-trimmed reads per sample was 3 193 777. There was a slightly higher average number of reads for domesticated *somniferum* than for wild *Papaver* accessions (table 1), but variation was detected for the different *Papaver* species. The number of quality-trimmed reads varied from 2 090 941 in accession W623843 (*P. orientale*) to 4 981 237 in accession PAP 834 (*P. somniferum*) (electronic supplementary material, S1). Four accessions (PI304971, C42, D52 and PI688271) missed over 95% of the SNPs detected for the whole set missing and were thus excluded. After applying quality filters to the 186 remaining accessions (no more than 10% missing data, MAF = 0.05 and coverage = 8×), Dataset1 retained 4102 SNPs, while Dataset2, comprising only *P. setigerum* and *P. somniferum*, included 7048 SNPs. A similar distribution of SNPs was detected in the 11 chromosomes both before and after quality filtering when comparing unfiltered data with Dataset1 (electronic supplementary material, S2).

**Table 1.** Statistics of genotype-by-sequencing and genetic diversity measures for taxa within the *Papaver* genus, populations defined by STURCTURE for the *K* = 5 model with the complete panel, and populations defined by STURCTURE for the *K* = 3 model with the *setigerum* and *somniferum* panel.

taxa	no. of accessions	quality trimmed reads	PiPerBP	thetaPerBP	*H*_E_	*H*_O_	Tajima's *D*	proportion of missing data
*Papaver armeniacum*	1	3 095 541	NA	NA	0.0090	0.0180	NA	0.4460
*Papaver* *dubium*	3	3 282 374	0.1290	0.1392	0.0748	0.0790	−0.1618	0.3490
*Papaver* *fugax*	1	2 676 265	NA	NA	0.0053	0.0105	NA	0.4670
*Papaver* *glaucum*	3	3 154 483	0.0203	0.0254	0.0181	0.0265	−0.2684	0.4920
*Papaver* *macrostromum*	1	2 974 736	NA	NA	0.0112	0.0224	NA	0.5530
*Papaver* *orientale*	8	2 629 207	0.1215	0.0836	0.0492	0.0234	3.1450	0.3360
*Papaver* *pseudo-orientale*	2	3 264 249	0.0168	0.0237	0.0132	0.0212	−0.3779	0.2690
*Papaver* *rhoeas*	4	3 019 826	0.0988	0.1451	0.0589	0.0536	−1.1907	0.4280
*Papaver* *rupifragum*	1	3 429 772	NA	NA	0.0017	0.0034	NA	0.4990
*Papaver* *somniferum* subsp. *setigerum*	19	3 374 131	0.1642	0.1237	0.1597	0.2211	1.3876	0.0120
*Papaver* *somniferum* subsp. *somniferum*	139	3 269 572	0.0381	0.0645	0.0379	0.0145	−1.3572	0.0040
*Papaver umbonatum*	4	2 753 721	0.0299	0.0429	0.0230	0.0239	−0.7949	0.5050

*K* = 5 model all accessions								
Pop1 (blue)	31	—	0.0230	0.0225	0.02247566	0.01386147	0.0870	—
Pop2 (red)	83	—	0.0290	0.0226	0.02872727	0.01293207	0.9738	—
Pop3 (black)	28	—	0.2438	0.1513	0.1550467	0.03128191	2.9714	—
Pop4 (brown)	15	—	0.1665	0.1209	0.1631392	0.286181	1.6856	—
Pop5 (purple)	29	—	0.0430	0.0496	0.04166388	0.01465319	−0.5230	—

*K* = 3 Setigerum and Somniferum								
Pop1 (red)	103	—	0.2481	0.1824	0.03357104	0.013454	1.2283	—
Pop2 (blue)	40	—	0.2076	0.1898	0.02823941	0.01355491	0.3530	—
Pop3 (brown)	15	—	0.1956	0.1989	0.1631392	0.286181	−0.0739	—

### (b) Population structure and phylogenetic inference

**Figure 1.**
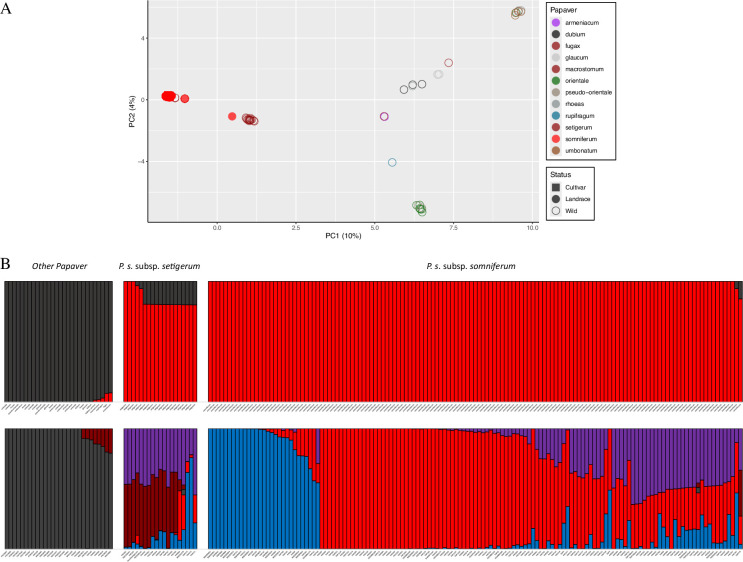
Population structure of 186 accessions of wild and cultivated *Papaver* based on 4102 SNPs (Dataset1) discovered by GBS. (A) PCA: wild accessions are shown as open points and cultivated *somniferum* as full points. (B) STRUCTURE *K* = 2 and *K* = 5 models.

Both the non-parametric PCA and the Bayesian STRUCTURE methods separated *somniferum* and *setigerum* from accessions of all the other species essayed (figure 1A,B). The effectiveness of the method is also attested by the clustering together of species *P. orientale* and *P. pseudo-orientale* in the PCA (lower right corner of figure 1A) and of the three *P. glaucum* accessions, as expected by taxonomical affinity. With the wild forms, some unexpected observations were made. For example, two accessions of *P. fugax* and *P. armeniacum* were genetically similar and a cluster (upper right corner of figure 1A) agglutinated plants of *P. umbonatum, P. rhoeas* and *P. orientale*. This suggests accessions mislabelling as no taxonomic similarity has been previously reported among these taxa [93]. In the phylogenetic analysis of Dataset1 (electronic supplementary material, S3) no wild *Papaver* other than *setigerum* seems to be genetically closer to the *somniferum* accessions analysed. Phylogenetic analysis shows the evolutionary distance between *P. somniferum* and other *Papaver* taxa (electronic supplementary material, S4A). The closest species to *P. somniferum* are *P. fugax* and *P. armeniacum.*

In the STRUCTURE analysis, Δ*K* indicated *K* = 2 and *K* = 5 as the most likely models. In the *K* = 5 model, all wild *Papaver* are clearly separated, whereas the cultivated poppy gene pool derives from three modelled clusters, all of which are represented in the *setigerum* accessions. This shows that none of the *Papaver* species assayed were involved in the domestication of *somniferum*. The highest genetic diversity in *Papaver* taxa (*H*_E_ and *H*_O_) was observed in *setigerum* with the lowest in *P. rupifragum* and *P. armeniacum* (table 1). *Somniferum* had the highest *F*_ST_ of all the taxa tested (electronic supplementary material, S4B).

**Figure 2.**
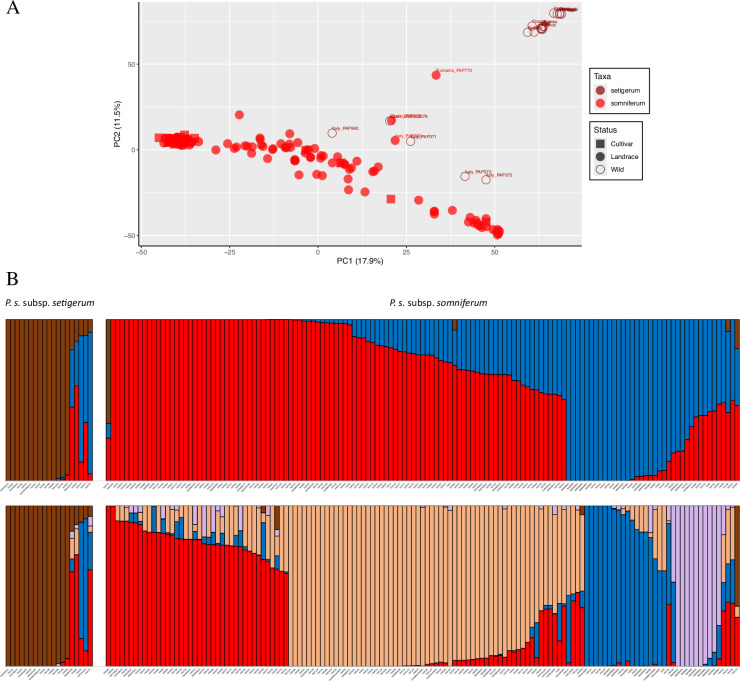
Population structure of 158 accessions of *Papaver somniferum* and *Papaver setigerum* based on 7048 SNPs (Dataset2) discovered by GBS. (A) PCA: accessions described as wild in the genebank passport data are shown as open points and those referred to as domesticated *somniferum* as full points; names of accessions referred to in the text are noted. (B) STRUCTURE *K* = 3 and *K* = 5 models.

When only *somniferum* and *setigerum* accessions are analysed (Dataset2), most wild accessions are separated from the domesticated ones (figure 2). Three Italian and one Spanish *setigerum* accessions are genetically closer to the domesticated group (PAP 843, PAP 373, PAP 371 and BGE 032578) in the PCA plot (figure 2A). The latter accession is identical to a cultivated *somniferum* also from Spain (PAP 400). As these came from different seed banks, it is unlikely this similarity results from mislabelling or gene flow during regeneration of collections. Likewise, *setigerum* PAP 371 and *somniferum* PAP 760 are genetically similar. Δ*K* indicates *K* = 3 and *K* = 5 as the most likely models in this dataset. In the *K* = 3 model of STRUCTURE, the four abovementioned *setigerum* accessions are seen as sharing the same gene pools as *somniferum* (blue and red in figure 2B) with only a small contribution of *setigerum* alleles (dark red). Similarly, the *somniferum* PAP 400 and PAP 760 have a small contribution of the *setigerum* gene pool. All other *setigerum* are quite distinct from the cultivated forms, with the PCA suggesting a separation between plants from France, Italy and Spain in one hand, and plants from Portugal. There is also a *somniferum* accession from Romania (PAP 770) that resembles a hybrid between *setigerum* and *somniferum*. The *somniferum* accessions are divided into two distinct groups (blue and red) along the principal component 1 (PC1) axis (electronic supplementary material, S5) with STRUCTURE suggesting a high degree of allele sharing between these two groups in some accessions (figure 2B). The phylogeny produced by MrBayes confirms the monophyly of cultivated *somniferum* and an early separation between Pop1 (red) and Pop2 (blue) (electronic supplementary materials, S5 and S6).

Pop3 (dark red), which includes all the bona fide *setigerum* accessions, has the highest genetic diversity measured by *H*_E_ and *H*_O_, whereas the two cultivated Pop1 and Pop2 have similar levels of diversity (table 1).

**Figure 3.**
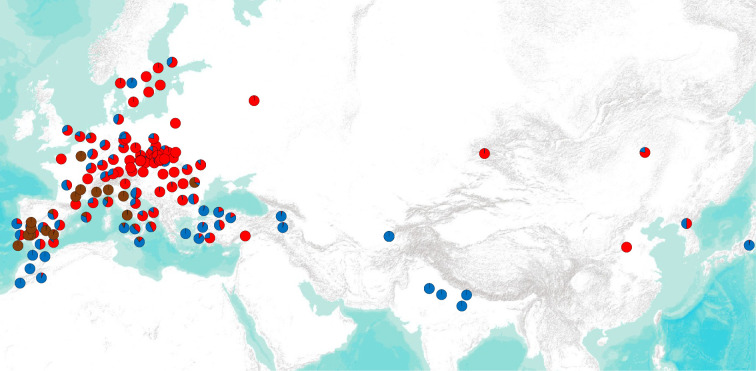
Geographical distribution of the three clusters identified in the STRUCTURE model *K* = 3 for *setigerum* and *somniferum* accessions. Each pie chart represents an individual accession and the proportional memberships to each of the three clusters.

The geographical distribution of the *K* = 3 populations shows that accessions throughout Europe receive their alleles from Pop1 (red), particularly the Central and Northern European ones, and the same is true of Chinese accessions (figure 3). Accessions clustered mostly in Pop2 (blue) are in smaller number and are more common in North Africa, south Europe, Turkey, Afghanistan, India and Japan. The geographical distribution of alleles derived from Pop3 (dark red) reflects the distribution of *setigerum* accessions and *somniferum* in their vicinity. The exception is a *somniferum* from Romania with a high proportion of alleles derived from Pop3. The geographical distribution of the sub-populations identified in the *K* = 5 model shows Pop2B (purple) encompassing accessions from India; Pop2A (blue) encompassing accessions from Turkey, North Africa and Italy, plus one accession from Scandinavia; Pop1B being predominant in East European and East Asian accessions and Pop1A being widely distributed in Europe (electronic supplementary material, S7).

### (c) Introgression and selection

The ABBA–BABA test detected introgression between *somniferum* Pop1 (red) and *setigerum* (*D* statistic: 0.2506; *Z* score: 4.4461). *Papaver orientale* was used as an outgroup (electronic supplementary material, S8). Those accessions considered hybrid or not clearly clustered in the population structure analysis were excluded. The Patterson’s *D* statistic allows the detection of introgression but not the quantification of how much of the genome is affected by it [94].

Both the GLM and MLM GWAS for the ‘domesticated’ trait in *P. somniferum* produced inconclusive results, with every chromosome having a series of SNPs associated with the trait (electronic supplementary material, S9). In the GLM GWAS, however, some outlier markers were observed in chromosomes 1, 3, 4, 7, 9, 10 and 11. BLASTING the sequences of the 20 SNPs with highest *−*log[10] *p*-values yielded an association with seven gene ontology annotated genes (electronic supplementary material, S10). These included, from the *P. somniferum* genome, an agmatine coumaroyltransferase-2-like protein gene (defence against pathogens in *Arabidopsis*), a deoxyloganetic acid glucosyltransferase-like (involved in the synthesis of monoterpene indole alkaloids accumulation) [95], a sodium/hydrogen exchanger 7-like gene (salt tolerance in soybean [96,97]), a chloroplast oxidoreductase, an AT-hook motif nuclear localized promotion factor gene (involved in a wide range of plant development processes [98,99]), as well as a methylenetetrahydrofolate reductase (lignin synthesis [100]) in *Brassica rapa*.

The GLM and MLM GWAS for seed colour detected SNPs associated with this trait in chromosomes 6 and 2, respectively, (electronic supplementary material, S11). One of these, in chromosome 6 of the *P. somniferum* genome is a 2-oxoglutarate dehydrogenase, shown to be involved in seed production, weight and number, as well as plant growth in *Arabidopsis* [101,102] (electronic supplementary material, S10). Another SNP detected by GWAS in chromosome 6 is associated with the phosphomethylpyrimidine synthase gene, involved in thiamine (vitamin B) biosynthesis, a compound that affects plant health, yield and immunity [103,104]. Moreover, an outlier SNP is located in the gene of the trifunctional UDP-glucose 4,6-dehydratase/UDP-4-keto-6-deoxy-D-glucose 3,5-epimerase/UDP-4-keto-L-rhamnose-reductase RHM1-like enzyme, which catalyses reactions in the cell wall synthesis and in flavonol biosynthesis [105,106]. Genetic diversity (π per base-pair) and Tajima’s *D* along the chromosomes was different between *setigerum* and *somniferum* for two of the zones of chromosome 6, between positions 54 and 62 000 000 and around position 190 000 000, where the markers detected by GWAS were positioned (electronic supplementary materials, S10 and S12). This suggests selection acting in these regions.

To investigate associations between allelic frequencies and environmental plus geographical variables (for those accessions with known provenance), a gradient forest method was used. To validate our analyses, we compared these results with those run from randomized matches between environmental data (while maintaining the geographical distance). The run with the original data performed better than the random counterpart (considering the accuracy importance data; electronic supplementary material, S13). However, no strong associations with the variables tested were found; the highest associations were with the components of the principal coordinates of neighbourhood matrix (PCNM—based on the Euclidean distance between geographical provenance of the accessions) (weighed *R*^2^ values approx. 0.01 in both runs). Likewise, the Mantel tests did not find strong associations between pairwise genetic distances and pairwise differences in geographical distances and environmental differences (electronic supplementary material, S14). The best correlation was with maximum temperature of the warmest month (*r* = 0.5379, *p* = 0.0001).

**Figure 4.**
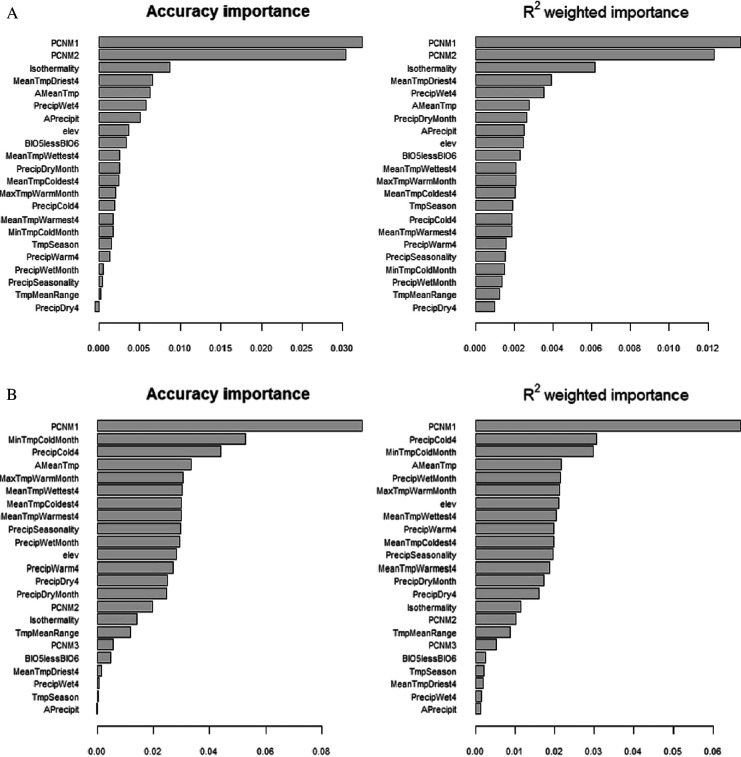
Association between environmental variables and allelic frequencies as well as between geographical and genetic distances, for the *K* = 3 model defined Pop1 and Pop2 (*setigerum* and *somniferum* accessions). (A,B) Random forest test for association between environmental variables and allele frequencies for Pop1 and Pop2, respectively.

To further investigate environmental associations with genetic differences, we repeated these analyses considering only the accessions in Pop1 (red) and Pop2 (blue), as defined by STRUCTURE and PCA (figure 2B and electronic supplementary material, S5; *K* = 3). For Pop1 (red), the Mantel test found very week correlations with insignificant *p* values (electronic supplementary material, S14), the highest of which (*r* = 0.1457) was with precipitation of driest quarter. Accordingly, the performance of both gradient forest runs (random and real data) was similar, with the highest association detected by the gradient forest between genetic data and PCNM1 and elevation having a weighed *R*^2^ values approximately 0.01 (figure 4A). For Pop2 (Blue), the Mantel test detected a significant high association with geographical distances (*r* = 0.7029) and the mean temperature of the coldest quarter (*r* = 0.549). The forest gradient test found a very weak association with PCNM1 (weighed *R*^2^ values < 0.01; figure 4B).

### (d) Seed geometric morphometrics

**Figure 5.**
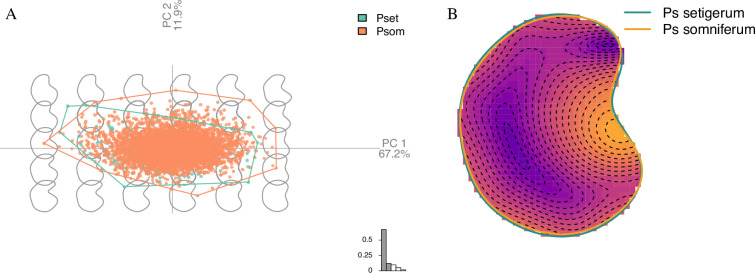
(A) GMM PC1 × PC2 factorial plane of the PCA on *Papaver* sp. shape variables. (B) Reconstructed mean shapes for the two poppy taxa shown with the intensity of differences between the two theoretical mean outlines.

The first PC of the PCA to investigate the overall shape of the seeds accounts for more than two thirds of the total shape variability (67.2%) and captures the depth of the hilum (figure 5A). The second PC accounts for 11.9% of the total shape variability and captures the skewness of the poppy seed (the asymmetry between the two parts above and below the hilum on the lateral view). The two taxa roughly occupy the same morphological space on the PC1 × PC2 factorial plane (figure 5B), and *somniferum* appears to have a slightly more variable shape occupancy of the global morphological space. This is confirmed by the reconstructed mean shapes (figure 5B).

**Figure 6.**
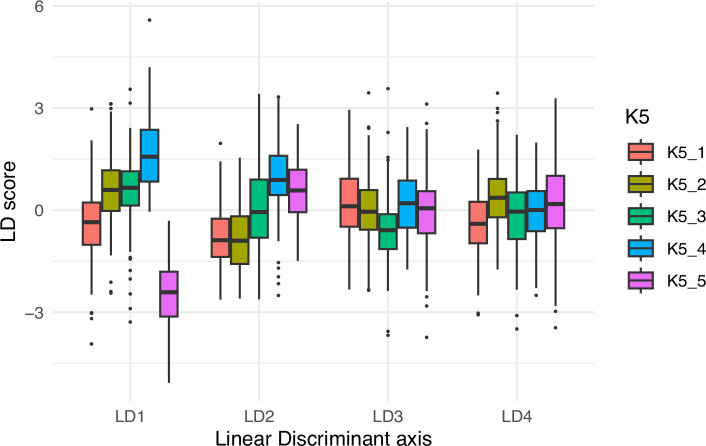
Scores on the four linear discriminant axes on a single and balanced (*n* = 150) LDA on the *K* = 5 grouping provided by STRUCTURE.

A single and balanced (*n* = 150) LDA was calculated on the SNP (*K* = 5) population attributions provided by STRUCTURE (figure 6). Class accuracies, the proportion of the 150 seeds correctly classified in their actual group, as obtained with leave-one-out cross-validation, are good for the K5_4 and K5_5 groups (i.e. Pop 2A and 2B, respectively) (>0.70) but below 0.5 for the three others. (electronic supplementary material, S15). This is reflected in the distribution of the scores of these groups onto the four linear discriminant axes of this single LDA (figure 6), which shows that LD1 clearly separates K5_5 (Pop2B) and, in a lesser extent K5_4 (Pop2A), from the other groups.

## 4. Discussion

### (a) Genotype-by-sequencing in *Papaver* accessions

The GBS method proved effective for inferring evolutionary relationships between cultivated *somniferum* and other species of the *Papaver* genus. The method was applied before to a panel of opium poppy accessions although this only included three *setigerum* [42]. We analysed a panel composed of *somniferum* accessions, a set of *setigerum* from this taxon’s distribution range and accessions of other wild taxa in the *Papaver* genus, to understand the domestication process. As a variant calling method, mapping of reads against the *P. somniferum* reference genome with FreeBayes generated a large amount of missing data in the final VCF file (table 1), particularly in individuals of taxa other than *P. somniferum* (44%). There was also three times more missing data in *setigerum* (1.2%) than in *somniferum* (0.4%) accessions, reflecting both the similarity and slight genomic distinctiveness of these two taxa. Despite the differences between species within *Papaver,* and the lack of a reference genome for species other than *P. somniferum,* we opt not to use other variant-call pipelines that work without a reference genome, such as STACKS [107]. Some literature suggests it is unlikely to improve the number of SNPs detected [108], and the VCF file generated has less annotations [109]. Moreover, the method used generated a high number of SNPs across all taxa, sufficient for the determination of population structure and other type of analysis. It must be noted that this reference genome bias might result in the underestimation of genetic diversity in the wild taxa or a distortion of divergence times in phylogenetic trees.

The excessive amount of missing data for four accessions (PI304971, C42, D52 and PI688271) can be explained by the taxonomic distance between *P. somniferum* and their species (*P. hybridum, P. occidentale* and *P. nudicale*) hindering mapping of reads to the *somniferum* reference genome or by technical issues during library preparation. A PCA with all the 190 accessions (based on a dataset containing no missing data with only 36 SNPs) hinted that these three taxa are in fact quite distinct from all the others (electronic supplementary material, S16).

PCAs computed on Dataset2 (assuming diploidy) and Dataset3 (assuming tetraploidy) for the *setigerum* and *somniferum* accessions did not show significant differences in population structure (electronic supplementary materials, S17A and S17B, respectively). This was a simple *in silico* way to address the fact that some *setigerum* accessions are described in the literature as tetraploid [7]. These data show that regardless of ploidy level, population structure and domestication history can be inferred from SNP data and are unaffected by differences in ploidy level. However, this might not be the case for other types of evolutionary analysis. The only noteworthy difference was *somniferum* accession PAP 770 (Romania) which was closer to the *setigerum* population than to *somniferum* in the diploid set but clustered with *somniferum* in the tetraploid set. It might be that this accession is a tetraploid. In the future, this will have to be confirmed by flow cytometry, as the accessions studied before [7] were not part of our panel. Replicates included in the panel (both from the same DNA extraction in different plates, and different plants within the same accession) displayed the expected genetic similarity that validates the method.

### (b) Wild progenitor and place of domestication of the opium poppy

In our panel, we included all *setigerum* accessions available in germplasm banks, and accessions of 13 other *Papaver* species, to see their relationships with the cultivated *somniferum*. PCAs, STRUCTURE and phylogenies all indicate that none of the 13 species tested is related to *somniferum* (figure 1A,B, electronic supplementary materials, S3 and S4). STRUCTURE also shows that there is no gene flow between these species and *somniferum,* although shared proportional membership to the modelled Pop3 (dark red) population is found between *setigerum* and *P. dubium*, *P. fugax* and *P. armeniacum* (figure 1B). These two latter taxa are also the ones suggested to be genetically closer to *P. somniferum* (including *setigerum*). In a recent study of phylogenetic relationships within the Papaveraceae family, based on two nuclear and seven plastid DNA regions, *P. armeniacum* is placed in a sub-clade within the *Papaver* 3 clade [93]. Likewise, *P. dubium* is also the phylogenetically closer taxa to *P. somniferum* in other studies [51,110–112], although the available literature is not consistent in this regard [49]. Our data are based on nuclear genome-wide markers, and it confirms these phylogenetic studies based mostly on chloroplast genomes and ribossomal DNA sequences. Importantly for the *Papaver* genus taxonomy, *P. orientale* and *P. pseudo-orientale* are genetically close, as well as *P. rhoeas* and *P. umbonatum*, and *P. fugax* and *P. armeniacum* (figure 1, electronic supplementary material, S4).

Our results argue against the hypothesis of *setigerum/somniferum* having originally evolved in the Fertile Crescent area, being introduced in Europe as weeds brought by the first farmers. They are genetically distinct from any of the *Papaver* species tested and, at least presently, *setigerum* is not found in the Fertile Crescent. Moreover, archaeobotanical evidence for *somniferum* seed remains appear in the Fertile Crescent only in later medieval contexts [9]. Of course, we cannot rule out the possibility that the wild progenitor of *somniferum* is indeed some other *Papaver* taxon native to the Fertile Crescent region that was not included in this study. However, based on the current archaeobotanical evidence we consider the ‘Western Mediterranean origin’ hypothesis as the most likely.

*Somniferum* and *setigerum* are clearly distinct from all the other *Papaver* species, but what is the relationship between them? Population structure analysis on Dataset2 shows they have very distinct gene pools, with the PCA showing a clear separation between *setigerum* accessions (PC1 and PC2 values above 50) relative to the *somniferum* accessions (figure 2). In this sense, and considering the whole panel population structure (where *somniferum* and *setigerum* form a cluster apart from other species), these two taxa are better described as subspecies of the same *P. somniferum* species. The exceptions to the distinct gene pool of these two taxa are the eight *setigerum* and *somniferum* accessions (named in figure 2) that stand intermediate to both clusters. Likewise, STRUCTURE models show these eight accessions share alleles from both the *setigerum* (dark red in figure 2B) and *somniferum* population (blue and red). Human error during library preparation is unlikely, as these samples were either in different plates or sections of the same plate. Replication in germplasm banks is also implausible. For example, according to the passport data, accession PAP 400 (*somniferum*) was collected by IPK in 1978 in Aranjuéz, near Toledo (Spain), whereas BGE032578 (*setigerum*) was collected by CRF on a roadside in Alcala de Henares. On the other hand, these two locations are only 50 km apart, so it is likely that these two accessions belong to the same population. Likewise, accession PAP 760 (*somniferum*) was collected in Calabria, Italy, in 1987, whereas accession PAP 371 (*setigerum*) was collected in 1979 near Palermo, Sicily. Could the seed banks have made a mistake in their taxonomical classification? Although not impossible, the two taxa have distinguishing features that should set them apart by a trained plant taxonomist. Previous comparisons of *setigerum* and *somniferum* based on genomic data by Hong *et al.* [42] did not find any outliers although they only included three *setigerum* accessions in their panel.

If we exclude errors, how do these eight accessions relate to domestication narratives? One possibility is that *setigerum* is indeed the sole wild progenitor of *somniferum*. The fact that this is the only taxa genetically closer to *somniferum* argues for this view. In this case, the outlier *setigerum* accessions can be descendants from the original wild population whose cultivation would lead to the domesticated *somniferum*. The fact they come from Italy and Spain, where archaeobotanical data suggest the earliest *somniferum* was cultivated, would support this. It is also noteworthy that two clusters of *setigerum* are evident in the PCA: one that includes accessions from Italy, France and Spain (closer to *somniferum*) and another (more distant from *somniferum*) with accessions from Portugal. The coincidence of the distribution range of *setigerum* with the earliest seed remains of *somniferum* in sites like La Marmotta, Le Taï or La Draga (all dated to 5500−4800 cal BCE) has long been pointed as an argument for the Western Mediterranean being the place of *somniferum* domestication [10]. The genetic proximity of *setigerum* accessions from these same regions to *somniferum* further strengthens the ‘Western Mediterranean origin’ hypothesis. Also, ABBA–BABA indicates a high level of introgression between Pop1 and *setigerum* (electronic supplementary material, S8), which could be the result of sympatry between the wild and domesticated forms, before the expansion of the cultivation range of *somniferum*. In this scenario, accessions in Pop1 probably represent the earliest domesticated forms and the area of distribution of *setigerum* acted as a hybrid zone where gene flow between wild and cultivated forms occurred. It is possible that this gene flow hindered the fixation of domestication traits in *somniferum* until landraces were established away from the natural distribution of *setigerum*, namely in Central and Northern Europe or in the Eastern Mediterranean. In these regions, full domestication and selection for specific traits would have occurred. Around Italy, Spain and south France, it would have been cultivated but the presence and introgression of wild *setigerum* would have delayed full domestication. This period of pre-domestication cultivation of wild and semi-domesticated forms, prior to the emergence of fully domesticated forms, has been proposed for other crops like wheat and barley [113].

The phylogeny produced with MrBayes (electronic supplementary material, S6) does not allow a clear picture of the relative divergence of Pop1 and Pop2. Phylogenetic methods to infer times of divergence for recent events, like domestication, are too imprecise in the absence of whole-genomes and other computational methods such as Globetrotter [114,115]. Seed morphometry indicating that *setigerum* is the only taxa within *Papaver* with seeds like *somniferum* [9] also supports the hypothesis of *setigerum* as wild progenitor. Although *setigerum* has much lower content of opiates than *somniferum*, the antispasmodic papaverine can reach high levels in *setigerum* [116]. *Setigerum* is the only taxon—other than *somniferum*—known to have the necessary metabolic steps for the biosynthesis of morphine and codeine [46,117]. It is possible that *setigerum* started to be cultivated as a medicinal plant, with a selection for varieties with higher levels of morphine and codeine occurring at a later stage. It is known that levels of BIAs can vary in *somniferum* depending on water stress, pathogenic agents or soil concentrations of zinc and cadmium [118–120]. Variations in these environmental factors may have originated *setigerum* populations that were richer in alkaloids, their growth and reproduction promoted by the first farmers in Western Europe.

An alternative hypothesis is that *setigerum* is not the progenitor of *somniferum* and that the latter was never domesticated, merely cultivated. As such, it never had a wild progenitor, and we cannot refer to a ‘domestication syndrome’. In this case, all *setigerum* would be feral forms of *somniferum* in different stages of ferality and genetic drift. Much literature reports that neither *setigerum* nor other *Papaver* species analysed have the high levels of opiates found in *somniferum* (morphine, codeine, thebaine, noscapine and papaverine) [23,121–127]. If the levels of bioactive alkaloids are so low in *setigerum,* it is difficult to explain how it got selected for cultivation and eventual domestication, especially for medicinal or mind-altering purposes. Unless, of course, *setigerum* was mainly used for food purposes or if the low alkaloid contents of *setigerum* were, nonetheless, mildly effective painkillers or narcotics. If, however, we assume that *somniferum* was already growing naturally in Western or Central Europe, the first farmers there may have discovered its properties, or learned them from local Mesolithic groups and cultivated it (no poppy seed remains have been recovered in Mesolithic contexts yet). A similar scenario has recently been proposed for another dual-purpose crop, *Cannabis sativa* (psychoactive drug and hemp fibre source), with no wild progenitor and with present-day hemp and drug varieties descending from an ancestral gene pool in East Asia that exists today as a mixture of feral plants and landraces [128].

The fact that both *setigerum* and *somniferum* possess the morphine synthesis pathway could be explained by *setigerum* being the wild progenitor (previous hypothesis) or, indeed, by the two taxa sharing a recent common ancestor. In this scenario, those outlier *setigerum* accessions would be feral forms: once cultivated *somniferum* that acquired a wild morphotype (e.g. PAP371 would be a feral form of PAP760). Feralization of opium poppy has been described not just in the Mediterranean area but also in locations where *setigerum* is unlikely to occur spontaneously, such as Korea or New Zealand [126,129–131]. In this case, those *setigerum* outlier accessions would be recent feralized forms of their genetically closer *somniferum* accessions (e.g. PAP371 would be a feral form of PAP760). The other bona fide *setigerum* would be a separate species that diverged recently from a common ancestor to *somniferum* (maybe through a polyploidization event, which would explain the tetraploid forms of *setigerum*). This is supported by the distinctiveness of the Pop3 (dark red) gene pool in STRUCTURE (figure 2). Moreover, the high number of differences that have been reported at the chloroplast genome level between *setigerum* and *somniferum* strengthens the case for them being two separate species [48].

Another possibility is that the outlier accessions are hybrids between *setigerum* and *somniferum*. Although considered a self-pollinator, *somniferum* is known to have varying degrees of outcrossing, including with *setigerum* [132]. This would explain why these eight accessions are separated from both the *setigerum* and *somniferum* clusters in the PCA. STRUCTURE suggests the accessions classified as *setigerum* are in fact *somniferum* introgressed by *setigerum* (they get most of their alleles from the *somniferum* blue and red populations), and that the Romanian accession PAP 770, on the contrary, is probably a *setigerum* introgressed by a *somniferum* (it gets most of its alleles from the *setigerum* ‘dark red’ population). This is a more likely scenario for those eight accessions, although it would fit both the ‘*setigerum* as wild progenitor’ and the ‘*setigerum* and *somniferum* as independent species’ hypotheses.

Future work will be needed to disentangle these scenarios. This will include (i) whole genome sequencing of *somniferum* and *setigerum* accessions; (ii) use of computational methods that disentangle hybrid swarms from feralization events; (iii) RNA-seq experiments to determine which genes in which metabolic pathways are exclusive to *somniferum*; (iv) field collections of *setigerum* plants for karyotyping alongside *setigerum* accessions held in germplasm banks. The latter should sample locations where *setigerum* has been described in disparate literature, but no records found (e.g. Canary Islands and North Africa) as well as putatively feral accessions from Korea and New Zealand. Reflecting the archaeobotanical data, we consider the hypothesis that *setigerum* is indeed the wild progenitor of *somniferum,* and that the outlier accessions are hybrids between *setigerum* and *somniferum,* to be the most parsimonious one.

### (c) Domestication syndrome in the opium poppy

The domestication syndrome is the suite of developmental, metabolic and phenotypic traits that differentiate a crop from its wild ancestor(s) [133]. In the case of opium poppy, this has rarely been studied, except for the difference in BIA levels and leaf shape [134]. In addition to the detection of genomic differences between *setigerum* and *somniferum,* we used GMM to investigate differences in the seed shape of the two taxa. Results showed that the morphometric differences between seeds of the *setigerum* and *somniferum* accessions shown here affected mostly the hilum area (figure 5), suggesting that the selective pressure associated with domestication of the opium poppy affected seeds (if we consider that *setigerum* is indeed the wild ancestor). It is unclear if this was a secondary effect of selection for some other traits or if the use of seeds (in culinary or oil production) led to selection for larger seeds.

Interestingly, GWAS also detected SNPs affected by domestication that are associated with seed weight and colour, supporting the suggestion that seeds were one of the organs most affected by domestication (assuming *setigerum* is indeed the wild progenitor). Other SNPs detected by GWAS are associated with cell wall synthesis and plant defence against pathogens, traits that have been recently discovered to be involved in the domestication process of other crops, such as barley and tomato [135,136]. Intriguingly, no gene involved in the synthesis of alkaloids was detected, suggesting that differences between *setigerum* and *somniferum* might be due not so much to the selection of genetic variants but to changes in the expression levels of particular genes. This has been shown for the domestication of cotton and maize [137–140]. One SNP was associated with a gene (deoxyloganetic acid glucosyltransferase-like) involved in alkaloid accumulation, reinforcing the idea that domestication involved changes in the level of specific alkaloids rather than the ability of them being synthesized. Further GWAS with alkaloid level phenotyping as well as an RNA-seq study comparing gene expression between low and high alkaloid content varieties (as well as wild *Papaver* accessions) could elucidate these issues.

### (d) Spread of opium poppy cultivation

We detected two major populations within *somniferum* each subdivided into two sub-populations (figure 2B). This agrees with a previous study on population structure of cultivated *somniferum* [42]. The geographical distribution of these populations likely reflects the spread of opium poppy cultivation. If so, there seems to have been two major stages in this process, which can also explain the separation of the two populations in the PCA plot (electronic supplementary material, S5). Pop1 is the most cosmopolitan and encompasses most accessions, including those that occur in the putative initial domestication area (Western Mediterranean). This can be interpreted as corresponding to the earliest cultivated stock that spread during the Neolithic period throughout Europe. This is supported by the fact that ABBA–BABA detects significant introgression between this population and *setigerum*, which does not happen with Pop2, despite the fact both populations have comparable levels of genetic diversity (table 1). Pop1B predominates in Eastern and Northern Europe, whereas Pop1A is more (broadly speaking) present in Western and Southern Europe. The fact that this geographical distribution is not clearly demarcated suggests that movement of cultivars occurred later. For example, accessions in China belong to Pop1B, and historical sources indicate it was introduced there during recent historical times [1]. The distribution of Pop2 in North Africa, Turkey, Afghanistan and India suggests this might correspond to accessions cultivated recently during the Arabic, Ottoman and Mogul periods, most likely for the purpose of obtaining opium [1]. All accessions from Afghanistan and India belong to Pop2B, as expected from a recent introduction of *somniferum* in this region. However, in the phylogeny shown in electronic supplementary material, S6, some Pop2 accessions seem to be ancestral to all the other *somniferum* and Pop1 is monophyletic. This might suggest that the separation between these two populations started early on during the domestication process, and it might just be that Pop2 accessions existed in the core domestication region in the past but are no longer found there. Two independent domestication events are not supported by the phylogeny or PCA analysis.

This geographical distribution cannot be explained by adaptation to different environmental regimes as the associations between allele frequencies and environmental variables were weak, both for the whole *somniferum* panel and for the two populations. This contrasts with other crops for which environmental adaptation played a role in shaping the geographical distribution of genetic diversity [141–144]. The absence of an environmental effect in *somniferum* genetic diversity might reflect a small-scale cultivation, practised mostly in gardens rather than in extensive rainfed fields, like cereals or some legumes. Nevertheless, results suggest that geographical distances and amount of rainfall have an effect—albeit a small one—on the distribution of genetic diversity in *somniferum*. The weak association observed between genetic diversity and environmental variables may be due to insufficient sampling or phenotyping, and this would have to be validated by common garden experiments [113].

Interestingly, there is also a correspondence between seed shapes defined by GMM and the clusters determined by STRUCTURE in the *K* = 5 model (figure 6 and electronic supplementary material, S15). GWAS also revealed SNPs associated with seed colour in chromosome 6. Could the (mostly abandoned) taxonomical categories of *somniferum, album* and *nigrum*, based on seed colour reflect the predominant use for opium production or culinary purposes [145]? Unfortunately, passport data for *somniferum* accessions do not indicate the end-use, context of its cultivation or opiate content, so this is difficult to infer. The study by Wong *et al.* [146] does not corroborate this hypothesis; though, from the six accessions with the highest codeine content, some had yellow others grey or black seeds. In addition, most *somniferum* accessions (but not *setigerum*) had high morphine contents regardless of seed colour.

The genomic and seed GMM data here help answer the questions proposed. (i) None of the most common *Papaver* species was the wild progenitor of *somniferum* or had any genetic contribution to its gene pool; *setigerum* was the wild progenitor or, also compatible with the data, *somniferum* does not have a wild progenitor, being the cultivated form of a naturally occurring species of which *setigerum* is a feral form. (ii) Considering the archaeobotanical data and the geographical distribution of genetic diversity in *somniferum*, the Western Mediterranean is the place where this species was originally domesticated (or cultivated); the hypothesis of an origin in the Fertile Crescent area followed by an introduction in Europe as a weed can be discarded. (iii) Phylogenetic analysis corroborates a single domestication wave, in the aforementioned region. (iv) After or during the domestication process, there were two major gene pool formation events in *somniferum*, the second of which is likely associated with the cultivation of varieties meant for opium production. (v) In addition to the higher content in BIAs, seed shape, cell walls and biotic defence mechanisms (and genes involved in these traits) were affected by the domestication process.

## Data Availability

The raw sequence data datasets were deposited in the BioProject database of NCBI [147]. The raw vcf file for Datasets 1 and 2 and raw data for the GMM analysis are available in [148]. Supplementary material is available online [149].
